# Gene model for the ortholog of *tgo* in *Drosophila mojavensis*

**DOI:** 10.17912/micropub.biology.001094

**Published:** 2025-08-06

**Authors:** Megan E. Lawson, Robert Mack Anderton Jr., Jorge Perez, Jacqueline Wittke-Thompson, Paula Croonquist, Chinmay P. Rele, Laura K. Reed

**Affiliations:** 1 The University of Alabama, Tuscaloosa, AL USA; 2 University of St. Francis, Joliet, IL USA; 3 Anoka-Ramsey Community College, Coon Rapids, MN USA

## Abstract

Gene model for the ortholog of tango ( *tgo* ) in the *Drosophila mojavensis* May 2011 (Agencourt dmoj_caf1/DmojCAF1) Genome Assembly (GenBank Accession: GCA_000005175.1 ). This ortholog was characterized as part of a developing dataset to study the evolution of the Insulin/insulin-like growth factor signaling pathway (IIS) across the genus *Drosophila* using the Genomics Education Partnership gene annotation protocol for Course-based Undergraduate Research Experiences.

**Figure 1. tgo gene model comparison between D. mojavensis and D. melanogaster orthologs:**
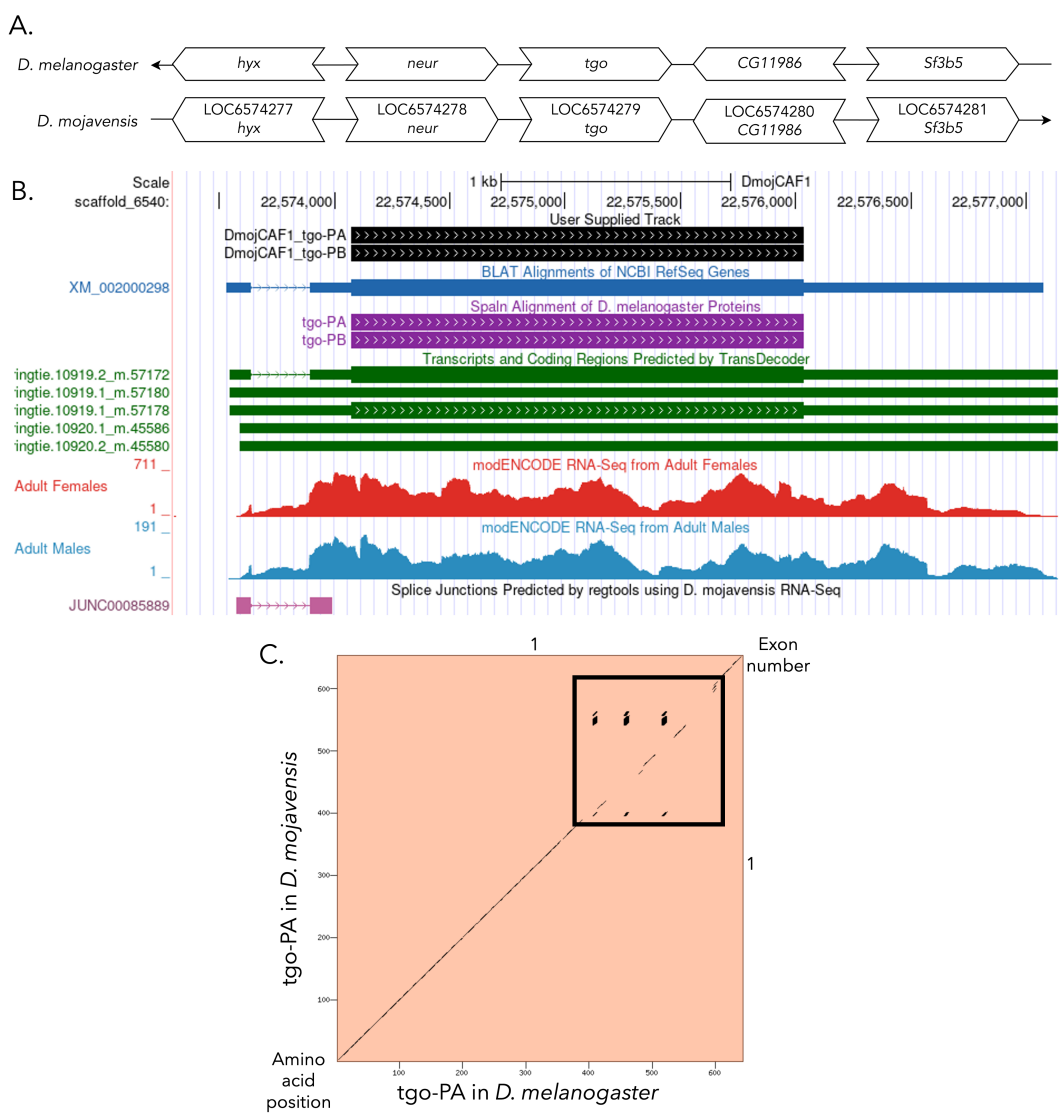
**
(A) Synteny comparison of the genomic neighborhoods for *tgo* in *Drosophila melanogaster* and *D. mojavensis* .** Thin underlying arrows indicate the DNA strand within which the reference gene– *tgo* –is located in *D. melanogaster* (top) and *D. mojavensis* (bottom). The thin arrow pointing to the left indicates that *tgo* is on the negative (-) strand in *D. melanogaster* , and the thin arrow pointing to the right indicates that *tgo* is on the positive (+) strand in *D. mojavensis* . The wide gene arrows pointing in the same direction as *tgo* are on the same strand relative to the thin underlying arrows, while wide gene arrows pointing in the opposite direction of *tgo* are on the opposite strand relative to the thin underlying arrows. White gene arrows in *D. mojavensis* indicate orthology to the corresponding gene in *D. melanogaster* . Gene symbols given in the *D. mojavensis* gene arrows indicate the orthologous gene in *D. melanogaster* , while the locus identifiers are specific to *D. mojavensis* . **(B) Gene Model in GEP UCSC Track Data Hub** (Raney et al., 2014). The coding-regions of *tgo* in *D. mojavensis* are displayed in the User Supplied Track (black); CDSs are depicted by thick rectangles and introns by thin lines with arrows indicating the direction of transcription. Subsequent evidence tracks include BLAT Alignments of NCBI RefSeq Genes (dark blue, alignment of Ref-Seq genes for *D. mojavensis* ), Spaln of *D. melanogaster* Proteins (purple, alignment of Ref-Seq proteins from *D. melanogaster* ), Transcripts and Coding Regions Predicted by TransDecoder (dark green), RNA-Seq from Adult Females and Adult Males (red and light blue, respectively; alignment of Illumina RNA-Seq reads from *D. mojavensis* ), and Splice Junctions Predicted by regtools using *D. mojavensis* RNA-Seq (SRP009365 - Chen et al., 2014). Splice junctions shown have a minimum read-depth of 10 with 100-499 supporting reads indicated in pink. **(C) Dot Plot of tgo-PA in *D. melanogaster* ( *x* -axis) vs. the orthologous peptide in *D. mojavensis* ( *y* -axis).** Amino acid number is indicated along the left and bottom; CDS number is indicated along the top and right, and CDSs are also highlighted with alternating colors. Line breaks in the dot plot indicate mismatching amino acids at the specified location between species. The boxed region highlights three glutamine tandem repeats present in the amino acid sequence of tgo-PA, along with several gaps in the alignment indicating insertions or deletions between the two species.

## Description

**Table:** 

* This article reports a predicted gene model generated by undergraduate work using a structured gene model annotation protocol defined by the Genomics Education Partnership (GEP; thegep.org ) for Course-based Undergraduate Research Experience (CURE). The following information in this box may be repeated in other articles submitted by participants using the same GEP CURE protocol for annotating Drosophila species orthologs of Drosophila melanogaster genes in the insulin signaling pathway. * "In this GEP CURE protocol students use web-based tools to manually annotate genes in non-model *Drosophila* species based on orthology to genes in the well-annotated model organism fruitfly *Drosophila melanogaster* . The GEP uses web-based tools to allow undergraduates to participate in course-based research by generating manual annotations of genes in non-model species (Rele et al., 2023). Computational-based gene predictions in any organism are often improved by careful manual annotation and curation, allowing for more accurate analyses of gene and genome evolution (Mudge and Harrow 2016; Tello-Ruiz et al., 2019). These models of orthologous genes across species, such as the one presented here, then provide a reliable basis for further evolutionary genomic analyses when made available to the scientific community.” (Myers et al., 2024). “The particular gene ortholog described here was characterized as part of a developing dataset to study the evolution of the Insulin/insulin-like growth factor signaling pathway (IIS) across the genus *Drosophila* . The Insulin/insulin-like growth factor signaling pathway (IIS) is a highly conserved signaling pathway in animals and is central to mediating organismal responses to nutrients (Hietakangas and Cohen 2009; Grewal 2009).” (Myers et al., 2024). “ *D.* *mojavensis* is part of the *mulleri complex* in the *repleta* species group within the subgenus *Drosophila* of the genus *Drosophila* (Wasserman 1992; Durando et al., 2000) *.* It was first described by Patterson (Patterson and Crow 1940). *D. mojavensis* specializes on rotting cactus as its host and is found in the Mojave and Sonoran Deserts of the southwestern United States and northwestern Mexico including the Baja Peninsula, as well as on the channel-islands off the coast of California (https://www.taxodros.uzh.ch, accessed 1 Feb 2023).” (Congleton et al., 2023).

We propose a gene model for the *D. mojavensis* ortholog of the *D. melanogaster* *tango* ( *tgo* ) gene. The genomic region of the ortholog corresponds to the uncharacterized protein XP_002000334.1 (Locus ID LOC6574279 ) in the May 2011 (Agencourt dmoj_caf1/DmojCAF1) Genome Assembly of *D. mojavensis* ( GCA_000005175.1 - Drosophila 12 Genomes Consortium et al., 2007). This model is based on RNA-Seq data from *D. mojavensis* ( SRP006203 - Chen et al., 2014) and *tgo* in *D. melanogaster* using FlyBase release FB2023_03 ( GCA_000001215.4 ; Larkin et al., 2021; Gramates et al., 2022; Jenkins et al., 2022).

The *Drosophila* *tango* ( *CG11987* , FBgn0264075, *tgo* ) gene encodes a bHLH-PAS protein that controls tracheal and CNS midline development (Sonnenfeld et al., 1997). Tango can form heterodimers with Single-minded and Trachealess, which bind a specific DNA enhancer element (Sonnenfeld et al., 1997), and *tango* mutants reveal defects in CNS midline and tracheal. In addition, the bHLH-PAS proteins Similar (Sima) and Tango are homologous to HIF-α and HIF-β, thus contributing to a conserved transcriptional response to hypoxia in *Drosophila melanogaster* (Lavista-Llanos et al., 2002). Insulin activates a HIF-dependent transcriptional response, which is mediated by the TOR and PI3K-AKT pathways. Sima's nuclear localization is increased when dAKT and dPDK1 are overexpressed, which mimics a hypoxic treatment (Dekanty et al., 2005).

***Synteny***

The reference gene, *tgo* , occurs on chromosome 3R in *D. melanogaster* and is flanked upstream by *neuralized* ( *neur* ) and *hyrax* ( *hyx* ) and downstream by *CG11986* and *Splicing factor 3b subunit 5* ( *Sf3b5* ). The *tblastn* search of *D. melanogaster* tgo-PA (query) against the *D. mojavensis* (GenBank Accession: GCA_000005175.1 Genome Assembly (database) placed the putative ortholog of *tgo* within scaffold 6540 ( CH933806.1 ) at locus LOC6574279 ( XP_002000334.1 )— with an E-value of 0.0 and a percent identity of 82.45%. Furthermore, the putative ortholog is flanked upstream by LOC6574278 ( XP_002000333.3 ) and LOC6574277 ( XP_015022843.1 ), which correspond to *neur* and *hyx* in *D. melanogaster* (E-value: 0.0 and 0.0; identity: 79.51% and 90.59%, respectively, as determined by *blastp* ; Figure 1A ; Altschul et al., 1990). The putative ortholog of *tgo* is flanked downstream by LOC6574280 ( XP_002000335.2 ) and LOC6574281 ( XP_002000336.1 ), which correspond to *CG11986* and *Sf3b5* in *D. melanogaster* (E-value: 0.0 and 8e-62; identity: 71.70% and 98.82%, respectively, as determined by *blastp* ). The putative ortholog assignment for *tgo* in *D. mojavensis* is supported by the following evidence: The genes surrounding the *tgo* ortholog are orthologous to the genes at the same locus in *D. melanogaster* and local synteny is completely conserved, supported by E-values and percent identities, so we conclude that LOC6574279 is the correct ortholog of *tgo* in *D. mojavensis* ( Figure 1A ).

***Protein Model***

*tgo* in *D. mojavensis* has one CDS within the genome sequence that is present in the two mRNA isoforms that differ in their UTRs (tgo-RA and tgo-RB), which are then translated into one unique protein-coding sequence (tgo-PA/tgo-PB; Figure 1B ). Relative to the ortholog in *D. melanogaster* , CDS and number of unique protein isoforms within the gene span are conserved. The sequence of tgo-PA in *D. mojavensis* has 77.58% identity (E-value: 0.0) with the protein isoform tgo-PA in *D. melanogaster* , as determined by *blastp* ( Figure 1C ). The protein sequences of tgo-PA in both species contain three glutamine tandem repeats and several insertions or deletions between the alignments, as is shown in the black box in Figure 1C . Coordinates of this curated gene model are stored by NCBI at GenBank/BankIt (accession BK065220 , BK065221 **)** . These data are also archived in the CaltechDATA repository (see “Extended Data” section below).

## Methods

Detailed methods including algorithms, database versions, and citations for the complete annotation process can be found in Rele et al. (2023). Briefly, students use the GEP instance of the UCSC Genome Browser v.435 ( https://gander.wustl.edu ; Kent WJ et al., 2002; Navarro Gonzalez et al., 2021) to examine the genomic neighborhood of their reference IIS gene in the *D. melanogaster* genome assembly (Aug. 2014; BDGP Release 6 + ISO1 MT/dm6). Students then retrieve the protein sequence for the *D. melanogaster* reference gene for a given isoform and run it using *tblastn* against their target *Drosophila* species genome assembly on the NCBI BLAST server ( https://blast.ncbi.nlm.nih.gov/Blast.cgi ; Altschul et al., 1990) to identify potential orthologs. To validate the potential ortholog, students compare the local genomic neighborhood of their potential ortholog with the genomic neighborhood of their reference gene in *D. melanogaster* . This local synteny analysis includes at minimum the two upstream and downstream genes relative to their putative ortholog. They also explore other sets of genomic evidence using multiple alignment tracks in the Genome Browser, including BLAT alignments of RefSeq Genes, Spaln alignment of *D. melanogaster* proteins, multiple gene prediction tracks (e.g., GeMoMa, Geneid, Augustus), and modENCODE RNA-Seq from the target species. Detailed explanation of how these lines of genomic evidenced are leveraged by students in gene model development are described in Rele et al. (2023). Genomic structure information (e.g., CDSs, intron-exon number and boundaries, number of isoforms) for the *D. melanogaster* reference gene is retrieved through the Gene Record Finder ( https://gander.wustl.edu/~wilson/dmelgenerecord/index.html ; Rele et al *.,* 2023). Approximate splice sites within the target gene are determined using *tblastn* using the CDSs from the *D. melanogaste* r reference gene. Coordinates of CDSs are then refined by examining aligned modENCODE RNA-Seq data, and by applying paradigms of molecular biology such as identifying canonical splice site sequences and ensuring the maintenance of an open reading frame across hypothesized splice sites. Students then confirm the biological validity of their target gene model using the Gene Model Checker ( https://gander.wustl.edu/~wilson/dmelgenerecord/index.html ; Rele et al., 2023), which compares the structure and translated sequence from their hypothesized target gene model against the *D. melanogaster* reference gene model. At least two independent models for a gene are generated by students under mentorship of their faculty course instructors. Those models are then reconciled by a third independent researcher mentored by the project leaders to produce the final model. Note: comparison of 5' and 3' UTR sequence information is not included in this GEP CURE protocol.

## Data Availability

Description: Zip file containing FASTA, PEP, and GFF. Resource Type: Model. DOI: https://doi.org/10.22002/b52a3-20p55
